# Time-dependent association between selective serotonin reuptake inhibitors and hospitalization due to hyponatremia

**DOI:** 10.1177/02698811211001082

**Published:** 2021-04-16

**Authors:** Buster Mannheimer, Henrik Falhammar, Jan Calissendorff, Jakob Skov, Jonatan D Lindh

**Affiliations:** 1 Department of Clinical Science and Education at Södersjukhuset, Karolinska Institutet, Stockholm, Sweden; 2 Department of Molecular Medicine and Surgery, Karolinska Institutet, Stockholm, Sweden; 3 Department of Endocrinology, Metabolism and Diabetes, Karolinska University Hospital, Stockholm, Sweden; 4 Department of Medicine, Karlstad Central Hospital, Karlstad, Sweden; 5 Department of Laboratory Medicine, Division of Clinical Pharmacology, Karolinska University Hospital Huddinge, Karolinska Institutet, Stockholm, Sweden

**Keywords:** SSRI, Syndrome of inappropriate antidiuretic hormone secretion, hospitalization, hyponatremia, sodium, adverse reaction

## Abstract

**Background:**

Selective serotonin reuptake inhibitors (SSRIs) have a wide and increasing use for the treatment of depression and anxiety. Previous studies have indicated an increased risk of hyponatremia during the first months of treatment. We aimed to investigate the detailed time-course of SSRI-associated hyponatremia with a high temporal resolution, using registry data encompassing the total Swedish population.

**Methods:**

This was a population-based case control study using several national registers. Patients hospitalized with a principal diagnosis of hyponatremia (*n* = 11,213) were compared with matched controls (*n* = 44,801). Multivariable regression was applied to explore time-dependent associations between SSRIs and hospitalization due to hyponatremia.

**Results:**

Individuals initiating treatment with SSRIs were exposed to an immediately increased risk for hospitalization at week 1, reaching an adjusted odds ratio (aOR) (95% confidence interval) of 29 (19–46). The associations then gradually declined, reaching an aOR of 2.1 (1.0–4.2) by week 13. The aOR for individuals treated for longer than 13 weeks was 0.78 (0.71–0.85).

**Conclusions:**

This study revealed a dramatically increased risk of hyponatremia exclusively related to newly initiated treatment. Consequently, even subtle symptoms consistent with hyponatremia during the first weeks of SSRI treatment should prompt analysis of sodium levels. In patients treated with SSRIs for several months or years, other causes should primarily be sought in the event of hyponatremia.

## Introduction

Hyponatremia is the most common electrolyte imbalance, affecting up to 30% of hospitalized patients (Upadhyay et al., 2006). Its clinical spectrum ranges from mild non-specific symptoms such as nausea and fatigue to severe symptoms such as confusion, seizures and even death (Mannheimer et al., 2019). One of the most common causes of hyponatremia is drug treatment (Adrogue and Madias, 2000). Thiazides (Filippone et al., 2020; Friedman et al., 1989), antiepileptics (Falhammar et al., 2018), antipsychotics (Falhammar et al., 2019b) and several other drugs have been linked to an increased risk of severe hyponatremia (Falhammar et al., 2019a; 2020). Selective serotonin reuptake inhibitors (SSRIs) are important for the treatment of depression and anxiety, with a widespread and increasing use (De Picker et al., 2014). Evidence suggests an important time-dependent association that may be exclusively related to a more recently initiated treatment (Coupland et al., 2011; Farmand et al., 2018; Leth-Moller et al., 2016). If confirmed, this knowledge may be important to differentiate a causal relationship between SSRI treatment and severe hyponatremia from a spurious association. Furthermore, detailed knowledge on the time-course is a prerequisite for an optimal strategy and an adequate communication in order to detect SSRI-induced hyponatremia. The aim of the current study was to investigate the time-course of SSRI-associated hospitalization due to hyponatremia in the Swedish population, with a high temporal resolution.

## Methods

This was a retrospective, population-based case-control study encompassing the adult Swedish population. Cases, defined as hospitalized subjects, aged 18 years or older, due to a first-ever primary diagnosis of hyponatremia (E87.1) or Syndrome of inappropriate antidiuretic hormone secretion (E22.2) between October 1, 2006 and December 31, 2014, were identified in the National Patient Register (NPR). A first ever diagnosis was defined as absence of a prior diagnosis (primary or secondary) of hyponatremia dating back to January 1, 1997. For each case, four controls without a previous diagnosis of hyponatremia were randomly identified using the Total Population Register. Controls were matched for age, sex and municipality. Each case was assigned an index date based on the date of hospital admission. For controls, the index date was defined as the index date of their matched cases. During the study period (January 1, 1997 to December 31, 2014), all diagnoses in the NPR were coded according to the International Classification of Diseases, 10th Revision (ICD-10). Concurrent and previous use of medications was identified using the Swedish Prescribed Drug Register (SPDR). The SPDR contains data on all prescribed and dispensed drugs in Sweden since July 1, 2005. Data on socioeconomic status was retrieved from the longitudinal integration database for health insurance and labor market studies register (LISA). The data collection process has been described in detail previously (Farmand et al., 2018). The study was approved by the Regional Ethical Review Board in Stockholm. Due to its retrospective epidemiological nature, no informed consent was required.

### Variables

SSRI dispensations were identified by Anatomical Therapeutic Chemical (ATC) codes starting with “N06AB”, including fluoxetine, citalopram, paroxetine, sertraline, fluvoxamine and escitalopram. Drug exposure was defined as a drug dispensation <90 days prior to the index date. If the first SSRI dispensation in the 90 days period was preceded by a one-year period without SSRI dispensations, the exposure was considered new, otherwise as ongoing. New exposures were further subdivided according to how many weeks (1–13 weeks) prior to the index date they had commenced. Confounding factors accounted for in the statistical analysis included concurrent medications, socioeconomic factors and medical conditions identified using information from the NPR, the SPDR and LISA. Cardiovascular events were subdivided into those occurring within 90 days prior to the index date and older events. A complete list of exposure and confounding variables is provided in Table 1.

**Table 1.** The definition of all factors included in the multiple logistic regression.

Variables	Codes
	ATC codes beginning with
Drugs of primary interest	
SSRIs	N06AB
Antiepileptic drugs	
Carbamazepine	N03AF01
Oxcarbazepine	N03AF02
Phenytoin	N03AB02
Valproate	N03AG01
Lamotrigine	N03AX09
Levetiracetam	N03AX14
Gabapentin	N03AX12
Antihypertensive drugs	
Thiazide diuretics	C03A, C09BA, C09DA, C03EA
Furosemide	C03C
Agents affecting the renin-angiotensin system	C09
Calcium channel blockers	C08, C07FB02, C09DB
Antibiotics	
Fluoroquinolones	J01MA
Macrolides	J01FA
Trimethoprim sulfamethoxazole	J01EE
Antidepressants	
Tricyclic antidepressants	N06AA
Other antidepressants	N06AX
Other drugs	
Amiodarone	C01BD01
Desmopressin	H01BA02
Proton pump inhibitors	A02BC, A02BD06
Lithium	N05AN
Antipsychotics (excluding lithium)	N05A excluding N05AN
NSAIDs	M01AA, M01AB, M01AC, M01AE, M01AG, M01AH, M01AX01, N02AJ08, N02AJ19
Statins	C10AA, C10BA02, C10BA03, C10BA05, C10BA06
	ICD10 codes beginning with
Renal diseases	
Renal insufficiency	N17–N19, procedure codes DR016, DR024, KAS00, KAS10, KAS20
Infections	
Sepsis	A41
Pneumonia	J18
Meningitis	G00–G07
Heart and vascular diseases	
Ischemic heart disease	I20–I25
New ischemic heart disease event^*^	I20–I24
Congestive heart failure	I50
Cerebrovascular diseases	I60–I64, I69
New cerebrovascular event^*^	I60–I64
Gastrointestinal diseases	
Pancreatic disease	K85, K860–K861
Inflammatory bowel disease	K50–K51
Liver diseases	K70–K77, procedure codes JJB, JJC
Other diseases	
Hypothyroidism	E03, E06.3
Malnutrition	E43.9, E41.9
COPD	J44
Pulmonary embolism	I26
Malignancy	C
	Combination of ATC- and ICD-10 codes, each beginning with
Alcoholism	ATC: N07BB03, N07BB04, N07BB01, N07BB05, N07BB ICD10: E244, F10, G312, G621, G721, I426, K292, K70, K860, O354, P043, Q860, T51, Y90-Y91, Z502, Z714
Adrenal insufficiency	ATC: H02AA, H01BA ICD10: E27.1, E27.2, E27.3, E27.4, E25
Diabetes mellitus	ATC: A10 ICD10: E10–E14
Socioeconomic factors	
Education	Increasing levels of education from 1 to 6, continuous variable
Income	Income in Swedish crowns during one year, continuous variable
Unemployment	Number of days, continuous variable
Proxy for frailty	
Drug use	Number of dispensed drugs 90 days prior to index date, categorized into <4, 4–7, 8–12 and >12 drugs
Duration of hospitalization	⩾3 days

COPD: chronic obstructive pulmonary disease; NSAIDs: non-steroidal anti-inflammatory drugs; SSRIs: selective serotonin reuptake inhibitors.

* During the 90 days before index date.

### Statistical analysis

The association between SSRI exposure and hyponatremia requiring hospitalization was investigated using univariable and multivariable logistic regression. In the primary analysis, the duration of exposure to SSRIs was stratified by weeks of exposure (1–13 weeks, >13 weeks), as described above. In secondary analyses, odds ratios (ORs) were calculated for SSRI exposure regardless of duration and separated into new (0–90 days) versus ongoing.

To quantitate the disease burden and absolute risk of SSRI-associated hospitalization due to hyponatremia, adjusted ORs (aORs) were used to calculate attributable risk percentages. The attributable risk percentage is defined as the percentage of cases in a population that is attributable to the exposure of interest. This statistic indicates the percentage of cases that could theoretically be prevented by removing the exposure from the population. *P*-values < 0.05 were considered statistically significant. All calculations were performed using R version 3.6.1.24.

## Results

During the study period, 11,213 individuals over 18 years of age were hospitalized with a principle diagnosis of hyponatremia (cases). In addition, 44,801 matched controls were included.

The majority were of female gender (72%) and the median age (range) was 76 (18–103) years. Table 2 shows a selection of medical characteristics and concurrent medications, including SSRIs, at index date among the study population. Overall, cases had more comorbidities and used more medications than controls. The most common accompanying medical conditions were previous malignant disease, ischemic heart disease and diabetes.

**Table 2.** Medical characteristics in addition to SSRI use among cases (individuals hospitalized due to hyponatremia) and matched controls at index date.

	Number of total cases (*n* = 11,213)	Number of total controls (*n* = 44,801)
Age, years (median, interquartile range)	76 (65; 84)	76 (65; 84)
Female gender	8074 (72.0%)	32,254 (72.0%)
Diagnosis		
Malignancy	3096 (27.6%)	9149 (20.4%)
Ischemic heart disease	2186 (19.5%)	6290 (14.0%)
Diabetes mellitus	1939 (17.3%)	5277 (11.8%)
Alcoholism	1764 (15.7%)	833 (1.9%)
Congestive heart failure	1453 (13.0%)	3541 (7.9%)
Cerebrovascular disease	1448 (12.9%)	3533 (7.9%)
COPD	1125 (10.0%)	1576 (3.5%)
Renal disease	489 (4.4%)	888 (2.0%)
Liver disease	421 (3.8%)	332 (0.7%)
Medications		
Antidepressants	2817 (25.1%)	5745 (12.8%)
Antiepileptic drugs	1061 (9.4%)	1128 (2.5%)
Furosemide	1735 (15.5%)	5487 (12.2%)
Calcium channel blockers	2283 (20.4%)	6432 (14.4%)
Betablockers	4175 (37.2%)	11,363 (25.4%)
Proxy for frailty		
Number of dispensed drugs 90 days prior to index date		
<4 drugs	2215 (20.0%)	22,892 (51.1%)
4–7 drugs	3421 (30.5%)	12,967 (28.9%)
8–12 drugs	3558 (31.7%)	7010 (15.6%)
>12 drugs	2019 (18.0%)	1932 (4.3%)
Previous hospitalization ⩾3 days duration	4852 (43.3%)	9477 (21.2%)
SSRI		
Total	1983 (17.7%)	3937 (35.1%)
Newly initiated^*^	695 (6.2%)	306 (2.7%)
Ongoing use	1288 (11.5%)	3631 (32.4%)

COPD: chronic obstructive pulmonary disease; SSRI: selective serotonin reuptake inhibitor.

* During the 90 days before index date.

**Figure 1.**
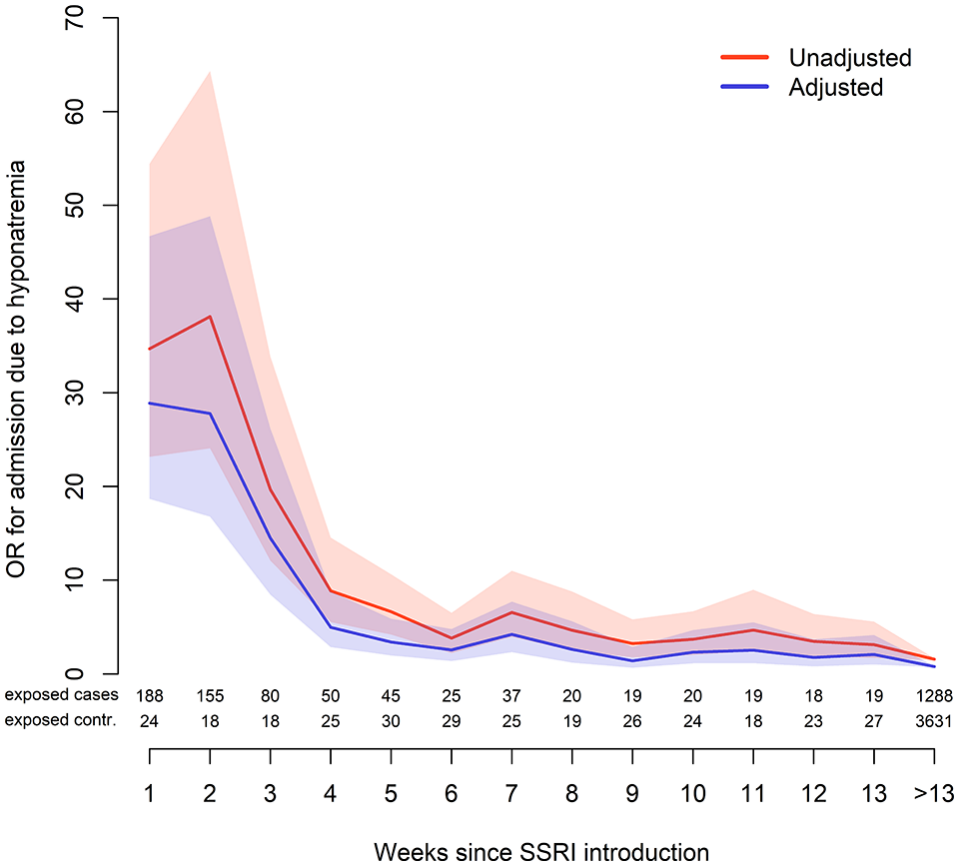
Unadjusted and adjusted ORs, accompanied with 95% CIs for hospitalization due to hyponatremia week by week.

The crude ORs (95% confidence interval (CI)) for hyponatremia among individuals exposed to SSRIs regardless of treatment duration was 2.23 (2.10–2.36). After adjustment for potential confounders, the OR was 1.22 (1.14–1.32). To further investigate the temporal association between initiation of SSRIs and hospitalization due to hyponatremia, we investigated the associations week by week (Figure 1). In general, crude ORs were higher than aORs. Individuals initiating treatment with SSRIs were exposed to an immediately increased risk for hospitalization at week 1, reaching an aOR (95% CI) of 29 (19–46). The associations then gradually declined, reaching an aOR of 2.1 (1.0–4.2) by week 13. The aOR for individuals treated for more than 13 weeks was 0.78 (0.71–0.85).

To estimate the corresponding disease burden, we analyzed the percentage of cases that were hospitalized due to hyponatremia potentially attributed to SSRI treatment (attributable risk percentage). For individuals with any SSRI exposure the attributable risk (95% CI) was 3.2% (2.1–4.2%), indicating that 363 individuals may have been hospitalized due to a hyponatremia attributed to SSRIs over the nine-year study period.

## Discussion

This is the first study specifically designed to investigate the time-course of SSRI-induced hyponatremia. It revealed an immediate dramatic increase in risk of hyponatremia sustained over the first four weeks that then gradually declined. By contrast, there was no evidence that ongoing treatment with serotonergic antidepressants increases the risk for hospitalization due to hyponatremia.

Although the association between SSRIs and hyponatremia has been known for many years, knowledge of the time-course has been insufficient (De Picker et al., 2014). Some studies have indicated a risk that is markedly higher (ORs ranging from 5 to 9) shortly after initiation as compared to ongoing treatment (Coupland et al., 2011; Gandhi et al., 2017; Leth-Moller et al., 2016). We have previously investigated the association between various antidepressants, including individual SSRIs and hospitalization due to hyponatremia. That study showed an effect that appeared to be exclusively related to newly initiated treatment (Farmand et al., 2018). For a more thorough investigation of the time-course of the risk, we have now analyzed week-by-week associations between the initiation of SSRIs and hyponatremia. Due to the greater resolution of the analysis focusing on individual weeks rather than a three-month period, as was the case in our previous paper, we now focused on SSRIs as a whole in order to reach sufficient power. Consequently, the present study was not able to inform on the risk associated with each individual substance. Although few studies have been able to compare the risk associated with individual substances, data indicate that the risk may be similar across different SSRIs (Coupland et al., 2011; Farmand et al., 2018; Gandhi et al., 2017). Also, for the reason of insufficient power, we chose not to investigate the effect of antidepressants that are less commonly prescribed in Sweden – for example, Monoamine oxidase inhibitors Tricyclic antidepressants, and Serotonin and norepinephrine reuptake inhibitors.

Results indicate an immediate and high increase in risk, sustained over the first four weeks, that then gradually declines (Figure 1). The mechanism for SSRI-induced hyponatremia has not been fully elucidated. However, animal studies point towards an underlying serotoninergic and α-adrenergic receptor mediated increase in antidiuretic hormone secretion (Brownfield et al., 1988; Leibowitz et al., 1990). We have previously presented time-dependent associations where the risk of severe hyponatremia is more pronounced in, or exclusively related to, newly initiated treatment with antihypertensive drugs (Falhammar et al., 2020), proton pump inhibitors (Falhammar et al., 2019c), opioids (Falhammar et al., 2019a) and antiepileptics (Falhammar et al., 2018). Hyponatremia following thiazide treatment may be the most well-known example that, according to experimental studies, can affect susceptible individuals within days of exposure (Filippone et al., 2020; Friedman et al., 1989). In a study with a similar setting and study design as the present one, we found that the risk for hospitalization was almost 60-fold increased during the first week of thiazide treatment and then gradually declined (Mannheimer et al., 2021). Although an increased risk for adverse effects seems plausible in the beginning of a new drug treatment, the mechanism with regard to the time-dependent risk associated with SSRIs remains unclear. This also accounts for the unexpected decreased association associated with long-term SSRI treatment. We can only speculate that factors, yet to be identified, predispose some individuals for severe hyponatremia, shortly after initiation of serotonergic treatment, resulting in a population more resilient to hyponatremia.

The study has important clinical implications. Firstly, it should prompt prescribing physicians to be attentive of symptoms consistent with hyponatremia when initiating SSRI treatment. Hyponatremia with SSRIs are known to occur among elderly females (Barber et al., 2015), as illustrated by the present study where the vast majority of cases were females (72%) with a median age of 76 years and a substantial frailty. Consequently, caution is particularly warranted among these groups of patients. Thus, even subtle symptoms occurring the first weeks after SSRI treatment initiation possibly indicating hyponatremia, should prompt measurement of sodium levels. Also important, the results of the current study support that the risk of hyponatremia is exclusively related to newly initiated treatment with SSRIs. Consequently, in patients with ongoing use of SSRI, recent onset of hyponatremia is unlikely to be coinciding with this treatment, and other causes should primarily be sought.

Finally, we found that SSRIs attributed to 3.2% of all individuals hospitalized due to hyponatremia during the study period. We have previously found that thiazides attributed to 27% of such cases (Mannheimer et al., 2021). Thus, the results further underscore drugs as a major cause of severe hyponatremia.

A major strength of the present study is its population-based design and the inclusion of all individuals with SSRI-associated hospitalizations due to hyponatremia in the Swedish population as a whole. The main limitation is the lack of available plasma sodium concentrations. However, since we only considered patients with a main diagnosis of hyponatremia, we made sure that only patients with clinically relevant hyponatremia were included. Clinically, the principal reason for hospitalization may be more adequate as compared to studies depending on hyponatremia as a secondary diagnosis, diagnoses made outside the hospital setting in the secondary or primary care (Coupland et al., 2011) or inclusion based on low sodium values, regardless of associated symptoms (Leth-Moller et al., 2016). To further motivate our study design, a validation of the principal diagnosis of hyponatremia was assessed. We found that 89% had been hospitalized mainly due to symptoms of hyponatremia. The mean plasma sodium concentration was 121 mmol/L – that is, profound hyponatremia – further emphasizing the clinical relevance of the study design (Farmand et al., 2018). Although hyponatremia as the main reason for hospitalization is a clinically relevant outcome, the focus of the current study may not include other relevant outcomes such as less severe hyponatremia. Another limitation of the present register-based study relates to the fact that adherence to oral medications is not complete in all patients. However, the most likely outcome of non-adherence is an under-estimation of the hyponatremia risk associated with SSRIs. Finally, while adjusting for a wide range of variables that may be related to hyponatremia, we cannot exclude the possibility of residual confounding.

In conclusion, this study revealed a dramatically increased risk of hyponatremia exclusively related to newly initiated treatment. Consequently, even subtle symptoms consistent with hyponatremia during the first weeks of SSRI treatment should prompt analysis of sodium levels. In patients treated with SSRIs for several months or years, other causes should primarily be sought in the event of hyponatremia.

## Supplemental Material

sj-docx-1-jop-10.1177_02698811211001082 – Supplemental material for Time-dependent association between selective serotonin reuptake inhibitors and hospitalization due to hyponatremia Click here for additional data file. Supplemental material, sj-docx-1-jop-10.1177_02698811211001082 for Time-dependent association between selective serotonin reuptake inhibitors and hospitalization due to hyponatremia by Buster Mannheimer, Henrik Falhammar, Jan Calissendorff, Jakob Skov and Jonatan D Lindh in Journal of Psychopharmacology
