# Supplementary material for: Time-dependent association between selective serotonin reuptake inhibitors and hospitalization due to hyponatremia
Source: J Psychopharmacol. 2021 Apr 16;35(8):928–33. doi: 10.1177/02698811211001082 (PMC8358543; doi:10.1177/02698811211001082)
Supplement: Supplementary file 1 [file sj-docx-1-jop-10.1177_02698811211001082.docx]

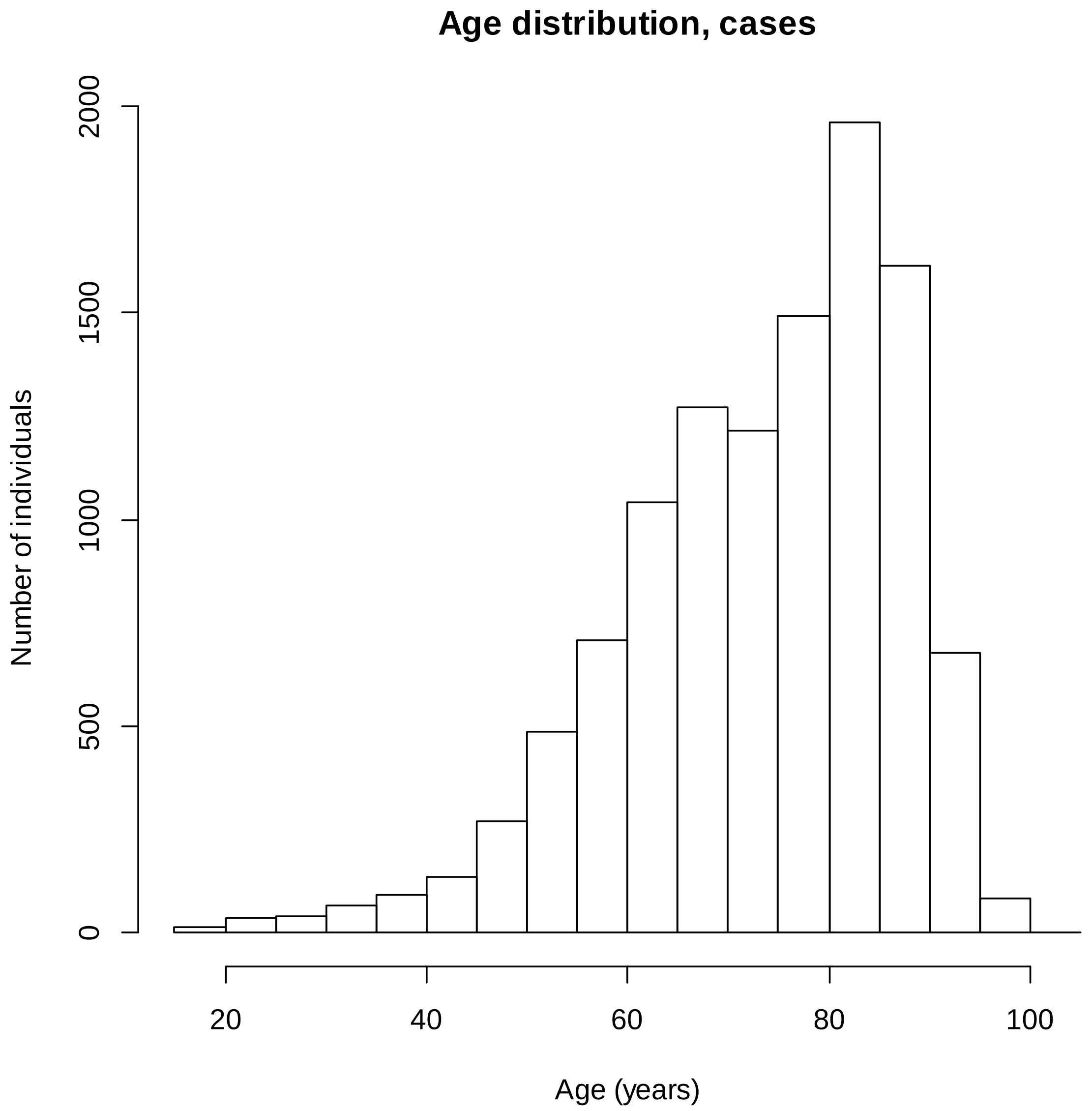

Age distribution, cases
Number of individuals
0
500
1000
1500
2000
20
40
60
80
100
Age (years)
